# Neonatal Genetic Screening Results for Spinal Muscular Atrophy in Romania: Insights from a 3-Years Pilot Program

**DOI:** 10.3390/ijns12010006

**Published:** 2026-02-01

**Authors:** Madalina Cristina Leanca, Gelu Onose, Georgiana Nicolae, Elena Neagu, Daniela Vasile, Ecaterina Bercu, Oana Mirabela Balanescu, Andrei Capitanescu, Constantin Munteanu, Cristina Popescu, Andrada Mirea

**Affiliations:** 1 Faculty of Medicine, University of Medicine and Pharmacy “Carol Davila”, 37 Dionisie Lupu Street, 020021 Bucharest, Romania; mada_mada332@yahoo.com (M.C.L.); geluonose@gmail.com (G.O.); dr.georgiananicolae@gmail.com (G.N.); andrada.mirea@gmail.com (A.M.); 2 Genetics Laboratory, National University Center for Children Neurorehabilitation “Robanescu-Padure”, 44 Dumitru Minca Street, 041408 Bucharest, Romania; elenaneagu1097@gmail.com (E.N.); danavd@gmail.com (D.V.); inabercu@yahoo.com (E.B.); t_oana92@yahoo.com (O.M.B.); 3 Teaching Emergency Hospital “Bagdasar-Arseni”, 12 Berceni Avenue, 041915 Bucharest, Romania; 4 Children’s Emergency Clinical Hospital “Maria Sklodowska Curie”, 20 Constantin Brancoveanu, 075534 Bucharest, Romania; andreicapitanescu@gmail.com; 5 Department of Biomedical Sciences, Faculty of Medical Bioengineering, University of Medicine and Pharmacy “Grigore T. Popa”, 700454 Iasi, Romania

**Keywords:** neonatal screening, spinal muscular atrophy, Romanian pilot program, genetic testing, early diagnosis

## Abstract

Spinal muscular atrophy (SMA) is a severe genetic neuromuscular disorder caused by bi-allelic deletions or pathogenic *SMN1* variants. Early diagnosis through neonatal screening is essential for timely therapeutic intervention, significantly improving clinical outcomes. In August 2022, a pilot neonatal screening program for SMA was launched in Romania, aiming to assess feasibility and impact. Objectives are to present the preliminary results of the ongoing SMA neonatal screening pilot program in Romania, evaluating its effectiveness in early detection and referral for treatment. The program started in August 2022 with four maternity hospitals and has progressively expanded to 28 maternity hospitals nationwide. Dried blood spot samples from newborns were analyzed for *SMN1* gene deletions using real-time PCR. Positive results were confirmed through genetic testing, and affected infants, along with their families, were referred for further medical evaluation and early therapeutic intervention. Approximately 60,000 newborns have been screened since the program’s inception, and 12 newborns tested positive for *SMN1* deletions, resulting in an estimated incidence rate of 1 in 5125 live births. All confirmed cases were promptly referred for specialized care, with early access to disease-modifying therapies. The program has faced challenges in logistics, parental awareness, and equitable access to treatment, but its expansion from 4 to 28 maternities demonstrates increasing feasibility, suitability, and acceptance. Conclusions: The Romanian pilot neonatal screening program for SMA has successfully identified affected infants early, proving its feasibility and clinical impact. The ongoing expansion suggests a strong foundation for a future national program, which could significantly improve early SMA diagnosis and patient outcomes in Romania.

## 1. Introduction

Spinal muscular atrophy (SMA) is a neurodegenerative genetic disease, with an average incidence of 1 in 10,000 live newborns [1], characterized by progressive and irreversible degradation of spinal motor neurons. Therefore, the motor function impairment has a major impact on the normal development of the child, with serious complications that affect motor skills and mobility, posture, nutrition, breathing, and cardiac function [2]. For the severe forms with the debut in the first six months of life, which represent more than half of the cases, late diagnosis and complications cause premature death [3]. This tragic situation makes SMA one of the main genetic diseases leading to death in the pediatric population [2]. Clinically, SMA is traditionally classified into types 0–4 according to age at onset and maximal motor function, with type 1 representing the most severe infantile form, and types 3–4 representing milder, later-onset phenotypes. This clinical spectrum is strongly influenced by the number of *SMN2* gene copies, which partially compensate for the loss of *SMN1*: infants with two copies are usually at highest risk for early-onset, severe disease (classically SMA type 1), those with three copies often present with intermediate phenotypes (type 2 or milder type 1), while individuals with four or more copies frequently develop later-onset, milder forms, corresponding to types 3–4, or may remain minimally symptomatic [4]. This genetic gradient underpins both the prognostic use of *SMN2* copy number and its role in guiding therapeutic urgency and choice in NBS-identified infants. The current availability of modern treatments with a decisively positive effect on the evolution of SMA has directed the efforts of the medical community towards detecting the disease as quickly as possible, so medical interventions have the effect of an absolutely normal development and life for the child with a genetic diagnosis of SMA [4]. Currently, many countries have started or are expanding newborn screening programs for SMA [5], yet some, like the UK, are still in the process of introducing it at the national level despite strong evidence supporting its clinical and cost-effectiveness [6]. This delay poses a significant impact on affected children, as untreated symptomatic SMA typically results in severe disability or death within the first two years of life [3]. Newborn screening enables timely access to transformative therapies such as nusinersen, onasemnogene abeparvovec, and risdiplam, which are now approved and funded in several health systems [7]. The introduction of SMA newborn screening is an urgent public health priority due to the severe consequences of delayed diagnosis and the availability of effective early treatments. It aligns with global trends in expanding genetic newborn screening and offers the chance to drastically improve the lives of children with SMA [8].

Romania has aligned itself with the general trend of optimizing the medical management of SMA by implementing standards of care (SoC) and by approval of disease-modifying therapies for current use [9]. However, the diagnosis is often delayed, the patients lose time, and, most importantly, motor neurons. In this case, their chances of having the maximum benefit from therapy and SoC are markedly decreased. For this reason, an early diagnosis through newborn screening is required [7]. In order to be part of the EU efforts to develop screening programs [10], a feasibility pilot study on newborn screening for SMA (NBS-SMA) started at the National Clinical Center for Children’s Neuropsychomotor Recovery “Robanescu-Padure” (Robanescu-Padure Center). The project has been continuously running since August 2022 and has been initiated and coordinated by the Robanescu-Padure Center. At first, 4 Bucharest maternities were included, then their number expanded to 28 (from Bucharest and from 10 different counties), thus our project has become a regional one.

## 2. Materials and Methods

### 2.1. Study Design and Participant Recruitment

This prospective pilot screening study was conducted from August 2022 to July 2025 across 28 maternity hospitals in Bucharest and 10 counties in Romania, coordinated by the Robanescu-Padure Center, Bucharest, Romania. Maternity personnel provided comprehensive information to parents regarding the screening process. Enrollment was contingent upon obtaining written informed consent from parents or legal guardians, with accurate recording of parental contact details. The study was conducted in accordance with the Declaration of Helsinki and approved by the Ethics Committee of the Robanescu-Padure Center (Protocol code 005/2022, approved on 16 Jun 2022).

### 2.2. Sample Collection and Transport

Blood samples for SMA screening were collected on specialized absorbent paper cards (dried blood spots, DBS) within the first 48–72 h after birth. Essential identification and collection details were registered directly on each card. The DBS cards, accompanied by corresponding informed consent forms (ICFs), were securely transported via accredited courier to the Robanescu-Padure Center one to two times per week. Upon receipt, Genetics Department staff verified concordance between DBS cards and ICFs, ensuring data integrity before registering all information into the Center’s informatics system. Unique laboratory codes were generated for each sample, providing full traceability throughout the screening and diagnostic workflow.

### 2.3. Screening Methodology

The workflow is based on a one-day protocol using a SMA simplex kit, commercially available, SALSA^®^ MC002 SMA Newborn Screen (MRC Holland, Amsterdam, The Netherlands) [11]. Dried blood spot (DBS) samples were processed using the CE-IVD-certified SALSA MC002 SMA Newborn Screen kit (MRC Holland, Amsterdam, The Netherlands). A single 3.2 mm punch from each DBS card was transferred into a 96-well PCR plate. DNA release from DBS was performed directly in the reaction wells according to the manufacturer’s protocol, without a separate extraction step, by incubating the punches in lysis buffer and subsequently adding the PCR master mix. The reaction mix contained primers and fluorescent probes specific for *SMN1* exon 7 and the internal reference targets supplied with the kit. Thermal cycling and melting-curve analysis were carried out on a real-time PCR platform compatible with high-resolution melting analysis using the cycling conditions recommended by MRC Holland, Amsterdam, The Netherlands. Fluorescence signals were recorded continuously, and post-run melt-curve profiles were analyzed with the manufacturer’s dedicated software; samples were classified as ‘*SMN1* exon 7 present’ or ‘*SMN1* exon 7 absent’ based on the shape and melting temperature of the amplification curves relative to kit controls. Each run included *SMN1*-deleted positive controls to ensure assay validity. All screen-positive or inconclusive results triggered repeat testing from a second DBS punch and subsequent confirmatory MLPA ((MRC-Holland, Amsterdam, The Netherlands) analysis on a freshly collected venous blood sample [12]. In the absence of a biochemical marker for SMA, only a genetic NBS-SMA technique detects the main cause of the disease, which is exon 7 complete absence (homozygous bi-allelic deletion) in the *SMN1* gene (~98% of cases). Various methods are applied for NBS-SMA worldwide, mostly based on real-time PCR. For our study we choose a CE-IVD method that fulfill several conditions: (i) it is suitable for our lab preexisting equipment; (ii) it is rapid, because there is no separate protocol for DNA extraction; (iii) it has great accuracy, with diagnostic sensitivity of 95–98%, diagnostic specificity ~100%, analytical sensitivity 100%, analytical specificity >95%; and (iv) it has been already used with success on large newborn populations (Poland, Serbia, and the Russian Federation). The SALSA^®^ MC002 SMA Newborn Screen kit is based on melt curve analysis (a standard PCR assay and incorporating fluorescence, followed by a melting protocol to distinguish the targets). The absence of the *SMN1* exon 7 detected by screening must be confirmed by the second-tier test, MLPA (MRC-Holland, Amsterdam, The Netherlands), which is the gold standard for SMA molecular diagnostics [13].

### 2.4. Confirmatory Testing

A positive test was communicated urgently by laboratory personnel to the SMA expert team at the Robănescu-Pădure Center. The team immediately informed the parents about the abnormal screening result, and a clinical consultation was scheduled as soon as possible, on the same day or the next day. The child was carefully examined, and a new blood sample was collected from the newborn for the second-tier confirmatory test, MLPA. After the result of the confirmatory test was received, the parents were invited to a second meeting to discuss the genetic diagnosis, receive genetic counseling, and decide on therapeutic options and initiation of standards of care for the genetically confirmed, presymptomatic SMA patient.

### 2.5. Statistical Method

Statistical analysis for the neonatal genetic screening pilot program in spinal muscular atrophy was conducted using SPSS (Statistical Package for Social Sciences, version 22.0) and Microsoft Excel 2010 [12]. The analysis included both descriptive and inferential approaches, assessing screening feasibility and accuracy [14]. The SALSA MC002 SMA Newborn Screen kit, based on a melting-curve analysis PCR assay, was used to detect *SMN1* exon 7 deletions, with confirmatory testing by MLPA. Assay performance metrics such as sensitivity, specificity, positive predictive value, and retest rates were calculated using established epidemiological methods. Additional key indicators—including the proportion of screening failures, coefficient of variability for replicate assays, and median time to result notification—were analyzed using parametric or nonparametric tests, depending on data distribution [11]. Outcomes were further correlated with clinical follow-up, evaluating the timeliness of treatment initiation and motor milestone acquisition in affected infants, to quantitatively assess program impact on health outcomes. Statistical significance was defined at *p* < 0.05 with 95% confidence intervals, ensuring robust and meaningful interpretation of results [15].

### 2.6. Clinical Follow-up and Referral Protocol

**Figure 1:**
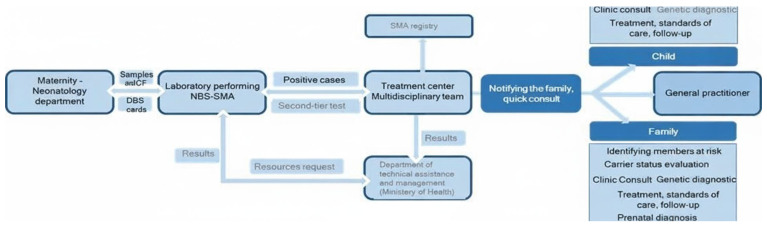
Key workflow steps for newborn screening and early management in spinal muscular atrophy (SMA). Maternity/neonatology units collect dried blood spot (DBS) samples after obtaining parental informed consent and send them to accredited laboratories for NBS-SMA testing. Suspected positive cases undergo a second-tier confirmatory test (multiplex ligation-dependent probe amplification, MLPA) and are referred to a multidisciplinary treatment center for clinical assessment and genetic confirmation. Families are rapidly informed of results and invited for clinical and genetic consultation, while results are communicated in parallel to relevant health authorities (for example, the Ministry of Health) for resource allocation and program oversight. The newborn is enrolled in structured follow-up with clinical specialists, and information is shared with the general practitioner for coordinated long-term care. Family members at risk are evaluated for carrier status, receive further genetic counseling, and are offered prenatal diagnostic options in future pregnancies. Confirmed cases are entered into a national SMA registry to enable long-term monitoring and research. Legend. Dark blue boxes indicate key clinical actors (maternity/neonatology units, laboratories, treatment centers, child, family), light blue boxes represent process steps and administrative bodies, and white arrows indicate the flow of samples and results along the screening–treatment pathway. Solid arrows represent the routine, forward workflow, whereas pale blue arrows indicate communication and feedback loops (results reporting, resource requests, and registry entry).

The workflow diagram for an NBS-SMA (Figure 1) pilot project illustrates the stepwise process from initial sample collection to post-diagnosis management for both the child and family. This ensures rapid detection, specialist referral, and comprehensive support. This streamlined workflow supports early intervention, rapid multidisciplinary involvement, and proactive family counseling—key features for effective SMA newborn screening pilot projects.

In addition to confirmatory testing in the newborn, parental carrier status was evaluated whenever possible. Following confirmation of a bi-allelic *SMN1* exon 7 deletion in the infant, both parents were offered molecular testing by MLPA, revealing that in 10 of the 12 families, both parents were healthy carriers of SMA. This assessment enabled genetic counseling regarding recurrence risk, cascade testing in extended family members, and options for prenatal or preimplantation diagnosis in future pregnancies.

## 3. Results

### 3.1. Screening Outcomes

**Figure 2:**
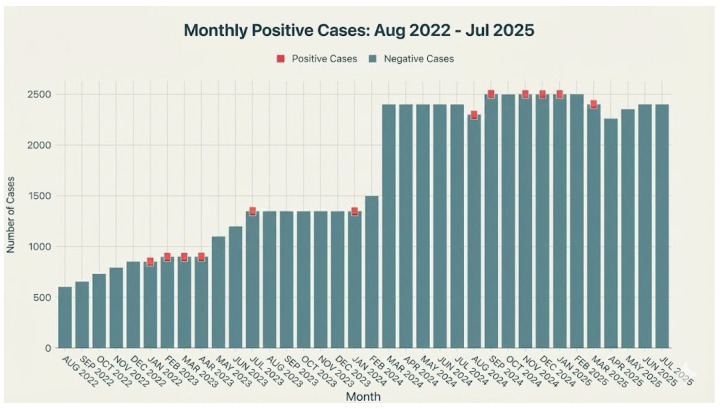
Distribution of tests performed and positive cases identified from August 2022 to July 2025.

Between August 2022 and July 2025, a comprehensive screening program was conducted, resulting in a total of 61,530 tests performed across a steadily expanding time frame. The monthly testing capacity grew significantly—from an initial 600 tests to a sustained peak of 2500 tests per month—demonstrating robust scalability and effective mobilization of resources. During this period, only 12 positive cases were identified, yielding an overall positivity rate of just 0.020%. Figure 2 presents a stacked bar chart illustrating the monthly distribution of tests performed and positive cases identified from August 2022 to July 2025. Each bar represents the total number of tests conducted in a given month, while the positive cases are distinctly highlighted in a vivid, contrasting color at the top of each bar and clearly labeled whenever they occur. The chart demonstrates not only the growth in testing capacity over the observed period but also underscores the sporadic and infrequent nature of positive cases despite the significant increase in monthly testing. Positive cases were rare and geographically dispersed, with no evidence of clustering in time or place. This pattern is consistent with the expected distribution of a rare autosomal recessive disorder in the general population, rather than with localized aggregation. These findings indicate both the high efficiency of the implemented screening protocols and a low prevalence of the targeted condition within the tested cohort, reinforcing the program’s public health value and its capacity to rapidly scale up without compromising the precision of case detection.

**Figure 3:**
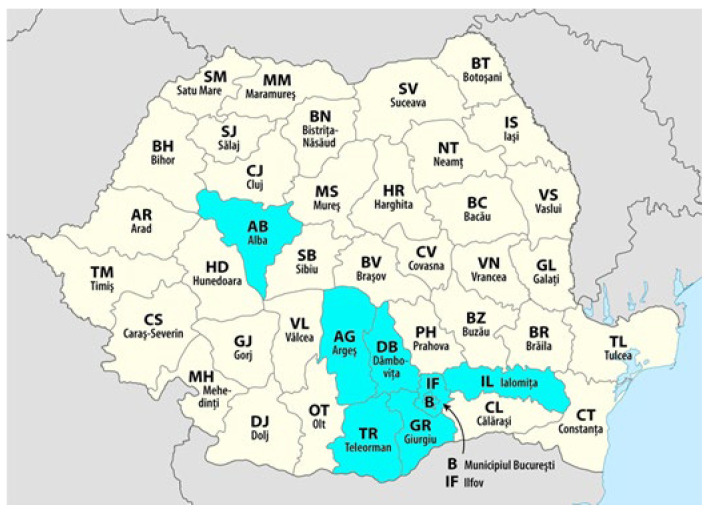
Regions with detected SMA-positive cases among newborns screened in the pilot program (August 2022–July 2025). Only counties participating in the pilot and with confirmed cases are shaded in blue.

Figure 3 illustrates the geographic distribution of confirmed SMA cases among newborns screened in the pilot program, which included maternity hospitals in Bucharest and 10 participating counties. Although not all Romanian counties were covered, this regional map provides preliminary epidemiological insight for targeted resource allocation, clinical surveillance, and stepwise expansion of screening within the currently participating areas and towards future national coverage. Counties with positive cases (București, Ialomița, Alba, Ilfov, Argeș, Dâmbovița, Teleorman, and Giurgiu) are labeled and colored in blue, while all other counties are displayed as zero. This visualization provides a clear geographical distribution of confirmed cases across the Romanian territory in this regional pilot study. By pinpointing locations with confirmed SMA cases, the map provides valuable epidemiological insight to guide resource allocation, clinical surveillance, and future expansion of screening programs across the country.

Among the 12 newborns diagnosed with spinal muscular atrophy (SMA) in the screening cohort, the median age at diagnosis confirmation was 22.9 days (SD 7.2, range 10–35 days), with most diagnoses confirmed within the first month of life (range: 10 to 35 days). Females accounted for a slight majority of cases (58%), and the largest number of diagnoses originated from the capital, Bucharest. In the cohort of newborns diagnosed with spinal muscular atrophy (SMA), analysis of the *SMN2* gene copy number revealed marked heterogeneity: 4 out of 12 cases (33%) had only two *SMN2* gene copies, typically associated with more severe disease phenotypes. Three children (25%) possessed three *SMN2* copies, and the largest subgroup—comprising five cases (42%)—had four copies, a configuration known to correlate with milder clinical forms and potentially better prognosis. These findings underscore both the promptness and geographic spread of early SMA detection and highlight the clinical diversity present in the molecular spectrum of affected Romanian newborns.

### 3.2. Treatment Decision-Making and Timing

Treatment decisions were made by the multidisciplinary SMA team according to national reimbursement criteria, *SMN2* copy number, clinical status at diagnosis, and parental preference. In general, infants with two or three *SMN2* copies and without contraindications were prioritized for onasemnogene abeparvovec when available, while risdiplam was selected in cases with higher *SMN2* copy number, existing comorbidities, or when gene therapy was not accessible. Of the 12 infants, five received onasemnogene abeparvovec and six risdiplam; one infant with four *SMN2* copies remained untreated at the time of analysis due to parental refusal. At the time of therapy initiation, 10 out of 12 infants were asymptomatic, while 2 presented clinical symptoms. Treatment was initiated at a mean age of 72.7 days (SD 45.4, range 17–150 days), with one case lacking treatment data. The average weight at treatment onset was 5.2 kg (SD 1.3, range 3.7–7.5 kg). These data underscore the effectiveness of early screening and intervention in this cohort, with timely therapeutic initiation and preferential use of genetic therapy or oral treatment. Figure 3 displays the distribution of ages (in days) at initial diagnosis and at first treatment for infants with spinal muscular atrophy (SMA) in the study cohort. It reveals that the age at initial diagnosis is generally low and tightly clustered (median and interquartile range around 20–30 days), reflecting efficient newborn screening and prompt clinical evaluation. In contrast, the age at first treatment shows a wider distribution, with a median above 70 days and a broader interquartile range (extending from approximately 40 to over 110 days). This indicates greater variability: some infants receive early intervention, while others experience longer delays before treatment, possibly due to logistical or clinical factors. The extended range in treatment age compared to diagnosis age underscores the ongoing need to identify and address barriers to rapid therapy initiation following early genetic diagnosis in SMA.

### 3.3. Motor and Developmental Outcomes

**Figure 4:**
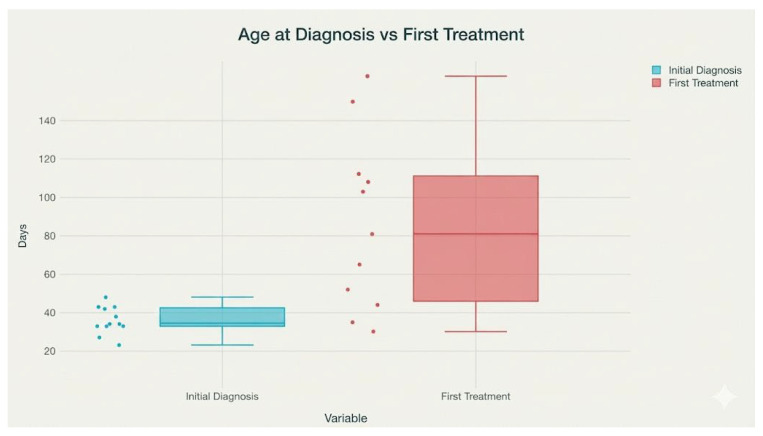
Variation in timing between early diagnosis and treatment initiation among SMA infants.

**Figure 5:**
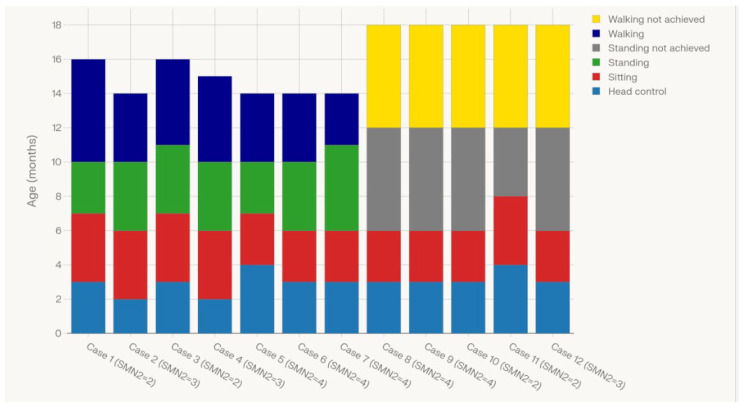
Variation motor milestone achievement by age and SMN2 copy number in SMA positive newborns.

Motor outcomes were assessed by recording the ages at which SMA patients achieved key motor milestones: head control, sitting without support, standing with support, and walking alone. The majority of children attained head control by age 3 months, with sitting achieved between 6 and 8 months. Standing with support was generally acquired between 10 and 11 months among children demonstrating advanced motor function, while independent walking was attained by a subset between 14 and 16 months. Nonetheless, most children attained early milestones such as head control and sitting, and no patient required respiratory support or feeding assistance at the time of evaluation, underscoring the importance of early detection and intervention in optimizing functional outcomes in pediatric SMA. Figure 4 comprehensively showcases the timing and pattern of motor development across the entire cohort, visually linking phenotypic heterogeneity to *SMN2* copy number and clinical trajectory. Figure 5 illustrates the ages at which individual SMA cases achieved successive gross motor milestones, stratified by SMN2 copy number, using a stacked bar representation of head control, sitting, standing, and independent walking. Each bar corresponds to a single case, with color segments indicating the age in months at attainment of each milestone and light-shaded segments denoting milestones that were not achieved by the time of last assessment. The figure shows that children with lower SMN2 copy numbers generally reached early milestones later and were less likely to achieve higher-order skills such as independent walking, whereas some children with higher SMN2 copy numbers attained standing and walking at comparatively younger ages and with more complete milestone acquisition across the spectrum.

#### 3.3.1. Two Copy SMN2 Group (*n* = 4 Infants)

All patients in the cohort received treatment at a median age of 20 days (range 10–25 days). Three patients were presymptomatic at the time of treatment, and all received onasemnogene abeparvovec, while one was symptomatic at treatment initiation with risdiplam. At the last follow-up, all patients who were presymptomatic at treatment remained asymptomatic. Among symptomatic infants at the time of treatment (*n* = 1), symptoms persisted at the last follow-up. The median initial head control was achieved at 3 months of age (range 3–4 months). Of the patients documented, two achieved sitting without support between 6 and 8 months, while three achieved standing with support (range 10–11 months), and two walked independently by 16 months of age. None of the patients required respiratory or feeding assistance. Of the patients with serial motor assessment, all demonstrated continued motor development without decline. Among the infants with two *SMN2* copies (*n* = 4 in this sample), motor milestones were generally achieved within expected ranges, with no cases of severe delay observed in this subset.

#### 3.3.2. Three Copy SMN2 Group (*n* = 3 Infants)

For the subgroup of patients with spinal muscular atrophy (SMA) carrying three copies of the *SMN2* gene, clinical data revealed notable early diagnosis and intervention patterns. Among these cases, all were diagnosed within the first month of life and exhibited rapid initiation of disease-modifying therapy, with ages at first treatment ranging from 22 to 137 days. In the three-copy subgroup, risdiplam was chosen for one infant because the parents preferred an oral therapy, whereas the other two met all criteria for onasemnogene abeparvovec, and the parents agreed with this treatment. None of the cases were symptomatic at the time of treatment initiation. Despite receiving early therapy, there was some phenotypic variability observed in motor milestone acquisition: all patients achieved head control and could sit unsupported by 6 months, but only the two patients treated with onasemnogene abeparvovec achieved independent standing or walking, whereas the risdiplam-treated patient had not attained these milestones at the last follow-up. Importantly, none of the patients required respiratory or feeding support at the time of evaluation. These findings underscore a favorable short-term outcome with early intervention in SMA patients with three *SMN2* copies, although some heterogeneity in motor progress remains.

#### 3.3.3. Four Copy SMN2 Group (*n* = 3 Infants)

In the subgroup of patients with four *SMN2* gene copies, early diagnosis and prompt therapeutic decisions were observed. All individuals were diagnosed within the first 35 days of life. Four out of five patients initiated treatment with risdiplam, while one case remained untreated at the time of evaluation. Only one patient showed symptoms at treatment initiation. Most patients commenced therapy within the first 150 days, and all treated individuals demonstrated robust achievement of developmental milestones: all attained head control, and the majority could sit unsupported by six to seven months. Three out of four treated patients achieved the ability to stand with support and walk independently by around 14 months, while two did not reach these milestones during the follow-up period. Respiratory and feeding support were not required in any case. These outcomes suggest that, in patients with four *SMN2* copies, early detection and proactive management with risdiplam result in favorable attainment of gross motor milestones, with minimal need for supportive interventions. However, a small proportion remains untreated or exhibits delayed progress, pointing to continued variability within this cohort.

## 4. Discussion

During the study period from August 2022 to July 2025, a total of 61,530 newborns underwent screening for spinal muscular atrophy (SMA). Among these, 12 cases of SMA were identified, corresponding to an incidence rate of approximately 0.0195%, or about 2 cases per 10,000 screened newborns. Females accounted for a slight majority of cases (58%), and this modest female predominance and the concentration of cases in Bucharest likely reflect random variation in a small sample and the higher birth volume and earlier inclusion of Bucharest maternities in the pilot, rather than a true sex-specific or regional difference in SMA incidence. This observed rate of new diagnoses reflects the expected rarity of SMA within the neonatal population. Furthermore, as the screening was conducted only once per individual and the entire tested cohort represents the observed population, the calculated prevalence of SMA within this group is likewise 0.0195%. These findings provide direct epidemiologic evidence for both the incidence and point prevalence of SMA in a large, prospectively screened population of newborns, aligning with international estimates and highlighting the value of systematic neonatal screening for early identification of this severe neuromuscular disorder. In comparison, internationally and in Romania, the general reported incidence of SMA is between 1 in 6000 and 1 in 10,000 live births, equating to rates of 0.017% to 0.010%, respectively. The prevalence is similarly low, due to the disease’s rarity and typically early onset in life. Local Romanian sources and international statistics both agree on this expected range for SMA incidence at birth (1/6000–1/10,000) and a carrier frequency of about 1 in 40 individuals for the responsible *SMN1* gene sequence variation. Thus, the incidence and prevalence rates observed in our screened cohort are very much in line with, or slightly above, the generally reported rates in Romania and globally, supporting the quality and representativeness of your population-based newborn screening effort.

The molecular profile revealed a distribution of two, three, or four *SMN2* gene copies, with the highest proportion (42%) carrying four copies, an important variable implicated in SMA phenotype severity. In large NBS cohorts from Germany and Southern Belgium, for example, infants with 2 copies typically represent the largest group, with those carrying 4 or more copies accounting for roughly one third to two fifths of cases [7,16], while data from New York State and multi-state US registries similarly show a smaller proportion of newborns with 4 or more copies compared with 2–3 copies [8]. The higher share of four-copy cases in our pilot may therefore reflect random variation in a small sample and the regional genetic background of the screened population and longer-term data with comparison to larger international cohorts will be needed to determine whether a genuine shift in SMN2 copy–number distribution exists in Romanian SMA patients or in the general population; this distribution nonetheless underscores the importance of genetic profiling in both prognostic assessment and therapeutic decision-making for infants identified through SMA newborn screening.

Patients with two *SMN2* copies represented the subgroup with the poorest baseline prognosis, as historically, this genotype is associated with more severe disease phenotypes. Nonetheless, in this cohort, early diagnosis and rapid initiation of gene therapy (onasemnogene abeparvovec) enabled the majority of patients to acquire essential motor milestones, such as head control, sitting, and even independent walking. Only the risdiplam-treated symptomatic patient showed delayed or incomplete achievement of these milestones. Importantly, none required respiratory or feeding support at follow-up, showcasing the benefit of pre-symptomatic or very early post-symptomatic treatment.

In the three-copy *SMN2* group, all patients were diagnosed and treated early, with a mix of onasemnogene abeparvovec and risdiplam usage. Similarly to the two-copy group, early therapy consistently enabled prompt acquisition of head and trunk control and sitting. The gene therapy-treated patients reached independent ambulation, but the risdiplam-treated patient lagged in motor milestone acquisition, suggesting a possible advantage of onasemnogene abeparvovec in this clinical context when administered early. None of the three-copy patients needed respiratory or feeding assistance.

The four-copy *SMN2* subgroup, expected to have the mildest phenotype, mostly received risdiplam, with one case untreated at the last follow-up. The predominance of risdiplam use among infants with four *SMN2* copies reflects the combination of a milder expected phenotype, evolving reimbursement frameworks, and the oral route of administration, whereas gene therapy was preferentially offered to those with 2–3 copies whenever feasible. The single untreated patient with four copies underscores persisting barriers such as family decision-making, despite early diagnosis. Despite this favorable genotype and overall earlier diagnosis, a degree of variability was still observed. While most achieved head control, sitting, and ambulation without respiratory or feeding support, two patients failed to reach standing or walking milestones at the time of analysis. This highlights that, although *SMN2* copy number is a reliable predictor of outcome, some heterogeneity persists even within groups considered at lower risk, potentially influenced by timing of intervention, initial clinical status, or other modifying factors.

These findings reinforce the paradigm that genetic stratification by *SMN2* copy number remains central to prognostication in SMA, but early identification through newborn screening and immediate initiation of disease-modifying therapy can substantially improve outcomes across all genotypes. In particular, onasemnogene abeparvovec appears associated with more complete achievement of major motor milestones when compared to risdiplam, especially in lower *SMN2* copy groups, though sample sizes are small. Even among patients with the most favorable genotype (four *SMN2* copies), delayed or absent treatment can result in suboptimal progress, indicating the continued importance of timely therapy.

No patients across any group required respiratory or feeding support, underlining the success of modern SMA management in preventing severe complications when intervention occurs in the presymptomatic or early symptomatic phase. Across our study and published pilot NBS outcomes, the need for respiratory and feeding support drops dramatically in pre-symptomatically treated patients. For example, the German SMARTCARE registry observed markedly less need for ventilatory or nutritional support compared to SMA infants diagnosed after symptoms [7]. Our cohort echoes this benefit, with zero subjects requiring such support, in line with international results.

The newborn screening pilot study in Romania using the SALSA^®^ MC002 SMA Newborn Screen kit [13] has demonstrated robust analytical accuracy, with no confirmed false positive or false negative results reported in the cohort. Each positive screening finding has been subsequently validated by confirmatory genetic testing, ensuring the reliability of case identification. The absence of misclassified results underscores the specificity and sensitivity of the implemented protocol, supporting its utility in early SMA diagnosis. This high level of diagnostic confidence has contributed to prompt clinical management and intervention for affected infants, reinforcing the value of systematic molecular screening in public health efforts targeting rare pediatric neuromuscular disorders. Continued surveillance and quality assurance remain essential to uphold these standards and monitor long-term screening performance as the program expands.

This study has several methodological limitations. The SALSA MC002 SMA Newborn Screen kit exclusively detects homozygous deletions of *SMN1* exon 7, which account for approximately 95–98% of SMA cases. Consequently, infants with compound heterozygous *SMN1* variants (one deletion and one non-deletion pathogenic variant) or with rare intragenic *SMN1* mutations may not be identified by this first-tier assay and could be missed by the screening program. Furthermore, the test does not distinguish *SMN1* carriers, and very low-level mosaicism or complex copy–number configurations might escape detection. Continuous quality assurance and long-term surveillance for clinically diagnosed SMA cases outside the screening pathway are therefore essential to monitor potential false-negative results and refine testing algorithms, for instance, by adding reflex sequencing in selected cases.

Recent European pilot studies—such as the SMARTCARE registry and screening projects in Germany [7] and Southern Belgium [16]—demonstrated that early, pre-symptomatic treatment enables most children with SMA (especially those with ≥3 *SMN2* copies) to achieve major motor milestones typically unattainable without early intervention. In the SMARTCARE registry, 90.9% of newborn-screened children with primarily two or three *SMN2* copies could sit independently, and 63.6% achieved independent walking, compared to just 14.7% in those diagnosed after symptom onset. Similarly, our data show that all treated patients with two or more *SMN2* copies achieved sitting, and a notable proportion achieved walking independently, especially if gene therapy was initiated promptly.

## 5. Challenges

Key challenges in newborn screening for SMA in Romania include limited financial resources, insufficient national funding, and competing healthcare priorities, which hinder the scale-up of screening programs. The implementation of SMA testing requires substantial investment in laboratory equipment, personnel training, and the development of logistical infrastructure—barriers that are especially pronounced in regions with constrained health budgets. Additionally, Romania faces one of the lowest rates of newborn screening coverage for rare diseases in Europe [17]. Organizational obstacles, such as the need for interdepartmental coordination, protocol adaptation, and ongoing professional training, further complicate the integration of SMA screening with existing newborn programs [6]. Despite these difficulties, pilot initiatives demonstrate that strategic planning and support can drive progress toward nationwide SMA screening coverage in Romania.

Beyond financial and organizational barriers, several additional challenges have affected the implementation of SMA newborn screening in Romania. Awareness of SMA and the benefits of early screening among both healthcare professionals and the general public remains low, which can hinder engagement and program uptake. Advocacy efforts are needed to educate stakeholders and policymakers about the impact of early diagnosis on patient outcomes [18]. The process of obtaining national policy endorsement and aligning SMA screening with existing public health regulations can be slow, resulting in delays in program rollout or expansion [19].

Disparities in healthcare infrastructure between urban and rural regions create logistical challenges for consistent sample collection, transport, and timely laboratory analysis, affecting equity of access to screening for all newborns.

Developing integrated data systems for tracking positive cases, ensuring timely follow-up, and facilitating referral to specialist care is essential but can be resource-intensive. Gaps in electronic health records and communication platforms may contribute to delays or missed diagnoses.

Reliable access to screening reagents, kits, and equipment is not always assured, particularly when procurement depends on external suppliers or limited budgets. Interruptions in supply chains can have direct consequences for program continuity. There is often a need for additional trained personnel—laboratory staff, genetic counselors, and pediatric specialists—with expertise in SMA and newborn screening, to ensure protocol adherence and effective patient management.

Addressing these multifaceted challenges requires ongoing commitment at national and regional levels, strategic partnerships, and investment in both human and technical resources to ensure sustainable and equitable SMA newborn screening across Romania.

## 6. Conclusions

The outcomes in our population are highly consistent with major international pilot studies: newborn screening with early initiation of effective therapies, regardless of *SMN2* copy number, transforms prognosis by supporting near-normal acquisition of milestones and minimizing severe complications [20]. Some heterogeneity in late gross motor skills and rare missed milestones persists, even in 3–4 copy patients, echoing observations from registry and trial data. These findings further reinforce the international consensus that early detection and prompt, genotype-guided therapy are fundamental to optimizing clinical outcomes in SMA [21].

In conclusion, while *SMN2* copy number strongly influences SMA severity, this effect can be significantly attenuated by early gene-targeted therapy, with contemporary treatments enabling near-normal motor development and minimal supportive care needs—even in traditionally high-risk genotypes [22]. Continued follow-up will clarify long-term outcomes and refine genotype–phenotype–treatment correlations in this era of transformative intervention. Romania also has plans and advocacy efforts underway for newborn screening of SMA to enable earlier diagnosis and treatment initiation, which is crucial for improving patient outcomes. These efforts are supported by strong advocacy from both national stakeholders and broader European initiatives. Organizations such as the European Alliance for Newborn Screening in Spinal Muscular Atrophy and SMA Europe are promoting the inclusion of SMA screening in all national panels across Europe by 2025, emphasizing the ethical imperative to detect the disorder as early as possible [23]. Early detection through screening enables access to disease-modifying therapies at a stage when efficacy is greatest, often before symptoms appear, resulting in significantly improved motor function and quality of life for patients. In summary, Romania’s progress in piloting and advocating for national SMA newborn screening brings the country in line with international trends, with the ultimate aim of diagnosing and treating SMA as early as possible to prevent irreversible loss of motor function and maximize patient outcomes.

## Data Availability

The data that support the findings of this study are available from the corresponding author upon reasonable request.
